# Molecular Characteristics of the Conserved *Aspergillus nidulans* Transcription Factor Mac1 and Its Functions in Response to Copper Starvation

**DOI:** 10.1128/mSphere.00670-18

**Published:** 2019-01-30

**Authors:** Zhendong Cai, Wenlong Du, Lianhong Liu, Daodong Pan, Ling Lu

**Affiliations:** a Jiangsu Key Laboratory for Microbes and Functional Genomics, Jiangsu Engineering and Technology Research Center for Microbiology, College of Life Sciences, Nanjing Normal University, Nanjing, China; b Key Laboratory of Animal Protein Deep Processing Technology of Zhejiang Province, College of Food and Pharmaceutical Sciences, Ningbo University, Ningbo, China; Carnegie Mellon University

**Keywords:** *Aspergillus fumigatus*, *Aspergillus nidulans*, Mac1, copper, transcription factor, transporter

## Abstract

Copper (Cu) is an essential trace element in all organisms, and Cu acquisition during periods of starvation is important for cell survival and proliferation. Although the Cu starvation-responsive transcription factor Mac1 as well as its targeted Cu transporters have been identified in Aspergillus fumigatus, the molecular mechanisms of Mac1-mediated Cu acquisition have not yet been investigated in Aspergillus. We demonstrated that Mac1 and its regulated Cu transporters are required for growth and conidiophore development during Cu starvation in Aspergillus nidulans. Moreover, A. nidulans Mac1 (AnMac1) showed highly functional conservation with the A. fumigatus homolog but not with homologs in Saccharomyces cerevisiae and Schizosaccharomyces pombe. Molecular characterization of Mac1 in A. nidulans demonstrated that the “Cu fist” motif (i.e., residues 1 through 40) harboring Cys, RGHR, and GRP residues is required for the Mac1-mediated low-Cu response but not the Cys-rich motifs REP-I and REP-II. Notably, overexpression of either the CtrA2 Cu transporter or the CtrC Cu transporter individually was unable to functionally rescue the defects in the AnMac1 deletion strain, implying that Cu uptake might require both CtrA2 and CtrC during Cu starvation, which is different from results seen with A. fumigatus. Findings in this study further suggest that the conserved Mac1-mediated Cu uptake machinery in A. fumigatus and A. nidulans is also species specific.

**IMPORTANCE** Copper is an essential cofactor of enzymes during a variety of biochemical processes. Therefore, Cu acquisition plays critical roles in cell survival and proliferation, especially during Cu starvation. Knowledge of the key motif(s) by which the low-Cu-responsive transcription factor Mac1 senses Cu is important for understanding how Cu uptake is controlled. Findings in this study demonstrated that the Cu fist motif, but not Cys-rich motifs, is essential for Mac1-mediated Cu uptake in Aspergillus. In addition, Cu transporters CtrA2 and CtrC are both required for Mac1-mediated Cu uptake during Cu starvation in A. nidulans, indicating that species-specific machinery exists for Cu acquisition in Aspergillus.

## Abstract (precis)

Copper is an essential cofactor of enzymes during a variety of biochemical processes. Therefore, Cu acquisition plays critical roles in cell survival and proliferation, especially during Cu starvation. Knowledge of the key motif(s) by which the low-Cu-responsive transcription factor Mac1 senses Cu is important for understanding how Cu uptake is controlled. Findings in this study demonstrated that the Cu fist motif, but not Cys-rich motifs, is essential for Mac1-mediated Cu uptake in Aspergillus. In addition, Cu transporters CtrA2 and CtrC are both required for Mac1-mediated Cu uptake during Cu starvation in A. nidulans, indicating that species-specific machinery exists for Cu acquisition in Aspergillus.

## INTRODUCTION

Copper (Cu) is one of the most abundant trace elements and is an essential nutrient for virtually all organisms (1–4). Consequently, Cu acquisition is important for cell survival and proliferation in low-Cu environments (5–8). In Saccharomyces cerevisiae, Cu uptake relies primarily on the low-Cu-responsive transcription factor Mac1-mediated high-afﬁnity Cu uptake system, which includes the Fre metalloreductases (Fre1 and Fre2) and Cu transporters Ctr1, Ctr2, and Ctr3 (9–12). In addition, stringent control of Cu uptake is also critical for avoiding excessive intracellular accumulation and toxicity (5). Under conditions of exposure to a high-Cu environment, S. cerevisiae Mac1 (ScMac1) is rapidly degraded, and that response is accompanied by decreased expression of the target Ctr transporters at both the transcriptional and posttranslational levels (13, 14). Molecular structure prediction analysis of ScMac1 revealed that residues 1 through 40, termed the “Cu fist,” function as the DNA-binding domain (15, 16). The Cu fist domain comprises of a Zn-binding motif (Cys-X2-Cys-X8-Cys-X-His) and a conserved (R/K) GRP sequence motif, both of which are essential for minor groove site-speciﬁc binding (6, 17, 18). In addition, ScMac1 encodes two Cys-rich motifs near its C terminus that are designated REP-I and REP-II, each containing five cysteines and one histidine residue (6, 19). However, the REP-I and REP-II motifs play distinct roles in ScMac1 function (13, 20, 21). The REP-I motif is essential for Cu sensing since site-directed mutagenesis experiments demonstrated that the REP-I motif is involved in elevating expression of the Ctr genes. Moreover, the REP-I motif can coordinate directly with Cu-positive (Cu^+^) ions (22). Although the REP-II motif can interact with Cu^+^ atoms, REP-II motif may alter the ability of ScMac1 to affect target gene expression but not its ability to sense Cu (13, 21, 22). In addition, ScMac1 repression induced by high concentrations of Cu arises from Cu-induced repression of intramolecular interactions between the Cu fist DNA-binding domain and the Cys-rich motif-containing activation domain (22). Notably, the Cu fist motif (but not the two Cys-rich motifs) is important for ScMac1 to function in the Cu-induced degradation of Ctr1 (14). Collectively, these lines of evidence suggest that both the Cu fist motif and the REP motifs are involved in S. cerevisiae Mac1-mediated responses to Cu levels.

Cu uptake mechanisms have also been identiﬁed in two human-pathogenic yeasts, Candida albicans and Cryptococcus neoformans, in which the Cu starvation-responsive transcription factor Mac1 and its homolog Cuf1, respectively, have vital roles in the regulation of Cu uptake (9, 23, 24). In addition, Ctr transporters, members of a high-afﬁnity Cu uptake system, possess conserved function across fungal species (25). Previous reports suggest that the C. albicans *ctr1* gene can functionally complement double deletions of S. cerevisiae *ctr1* and *ctr3* (26), suggesting that Cu acquisition mechanisms are conserved between the two genera. Previously, we and other groups demonstrated that the Cu-sensing transcription factor Mac1 coordinates with the Ctr transporter to regulate Cu acquisition in response to low-Cu environments in the human-pathogenic fungal species Aspergillusfumigatus (27–30). In addition, bioinformatics analysis showed that ascomycetes fungi possess signatures (including the “Cu fist” domain and the Cys-rich motifs at the N and C termini of Mac1) that are common features for the Cu starvation-sensing transcription factor (27). This finding indicates that these conserved motifs may be of importance in responding to extracellular Cu concentrations. However, the functions of the Cu fist and Cys-rich motifs in Mac1 and its Cu acquisition machinery have yet to be determined in *Aspergillus* spp.

Here, we demonstrated that Mac1 homologs in A. nidulans and A. fumigatus have a conserved function in response to low Cu levels. Further, our experiments indicated that a conserved Cu fist motif (but not the Cys-rich motifs) is essential for Mac1-mediated Cu uptake in A. nidulans. Moreover, species-specific machinery exists for the Mac1-Ctr Cu uptake pathway in A. nidulans and A. fumigatus.

## RESULTS

### Functional conservation of low-Cu-responsive transcription factor Mac1 in selected fungal species.

**FIG 1:**
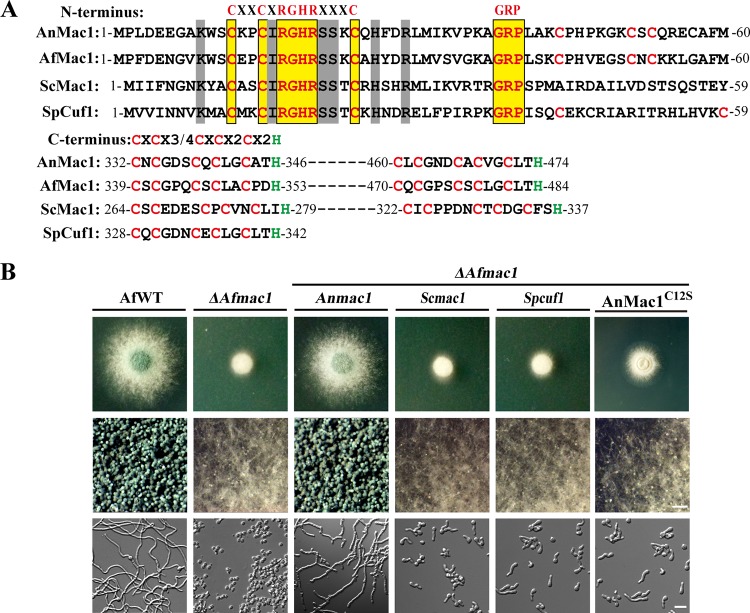
Functional complementation in the *ΔAfmac1* mutant (A) Schematic view of the AfMac1 homologs showing the Cu fist domain and Cys-rich motifs at the N and C termini. The sequences of AfMac1 homologs were aligned by using the AlignX module of Vector NTI Advance software. (B) (Top row) Phenotypic characterization of the strains grown on MM media with 1 μM Cu at 37°C for 40 h. (Middle row) Closeup views of the center of individual colonies taken with a stereo microscope. Scale bar = 100 μm. (Bottow row) Mycelia of the strains grown in liquid MM media (1 μM Cu) at 37°C for 10 h. Scale bar = 20 μm.

All selected Mac1 homologs contain an N-terminal Cu fist domain and C-terminal Cys-rich motifs, each containing five cysteines and one histidine residue (Fig. 1A). This similarity in protein architecture led us to hypothesize that A. fumigatus Mac1 (AfMac1) homologs could functionally replace each other. In order to test this hypothesis, we performed homolog replacement assays in the A. fumigatus *ΔAfmac1* background strain. The complementation strains were generated by individually introducing the A. nidulans *mac1* (*Anmac1*), S. cerevisiae *mac1* (*Scmac1*), and Schizosaccharomyces pombe *cuf1* (*Spcuf1*) genes, under the control of the *Afmac1* native promoter, into the *ΔAfmac1* mutant. As shown in Fig. 1B, introduction of *ΔAfmac1*^AnMac1^ (CZD01) restored the *ΔAfmac1* strain to the wild-type phenotype under conditions of Cu starvation stress, suggesting that AnMac1 can functionally complement AfMac1. In contrast, strains complemented with *Scmac1* and *Spcuf1* (strains *ΔAfmac1^Scmac1^* and *ΔAfmac1^Spcuf1^* [CZD02 and CZD03]) still exhibited the *ΔAfmac1*-like phenotype, with sparse conidia and shortened hyphae, under conditions of Cu starvation. Furthermore, semiquantitative reverse transcription (RT)-PCR analysis demonstrated that the two yeast homolog replacement strains exhibited comparable expression levels of *Scmac1* or *Spcuf1* in the background of the *Afmac1* gene deletion strain, suggesting that *Scmac1* and *Spcuf1* were normally expressed in the *Aspergillus* system at the mRNA level (see Fig. S1 in the supplemental material). The data indicate that, unlike the yeast Mac1 homologs, A. nidulans Mac1 may have a conserved function similar to that of the corresponding A. fumigatus homolog under low-Cu conditions.

10.1128/mSphere.00670-18.1 FIG S1 Veriﬁcation for the *Scmac1* and *Spcuf1* mRNA expression patterns in the homolog replacement strains. Semiquantitative RT-PCR analysis was performed with primer pairs Afmac1-F/Afmac1-R, Scmac1-F/Scmac1-R, and Spcuf1-F/Spcuf1-R for the A. fumigatus WT, *ΔAfmac1*^ScMac1^, and *ΔAfmac1*^SpCuf1^ strains, respectively. The *tubA* gene was used as an internal control, and rRNA data are shown as a control for RNA quality without the definite quantity. Download FIG S1, TIF file, 0.2 MB. Copyright © 2019 Cai et al. 2019 Cai et al. This content is distributed under the terms of the Creative Commons Attribution 4.0 International license.

We next investigated the function of the conserved Cys residues that are part of the homologous structures in both the Cu fist domain and the Cys-rich motifs in Mac1 homologs (Fig. 1A). A mutant AnMac1 variant was created by modifying the first Cys residue of AnMac1 at position 12 to Ser and then introduced it into the *ΔAfmac1* mutant. The resulting strain was referred to as CZD04 [*ΔAfmac1*^AnMac1(C12S)^]. In contrast to the wild-type strain, the Cys-mutated AnMac1 strain exhibited *ΔAfmac1*-like defects with shortened hyphae and reduced production of conidia, as observed microscopically, suggesting that the Cys residue at the position 12 is required for the normal function of AnMac1 during Cu starvation (Fig. 1B). This further underlines the potential importance of Cys residues within AnMac1.

### AnMac1 is responsible for hyphal growth and conidiophore development during Cu starvation.

**FIG 2:**
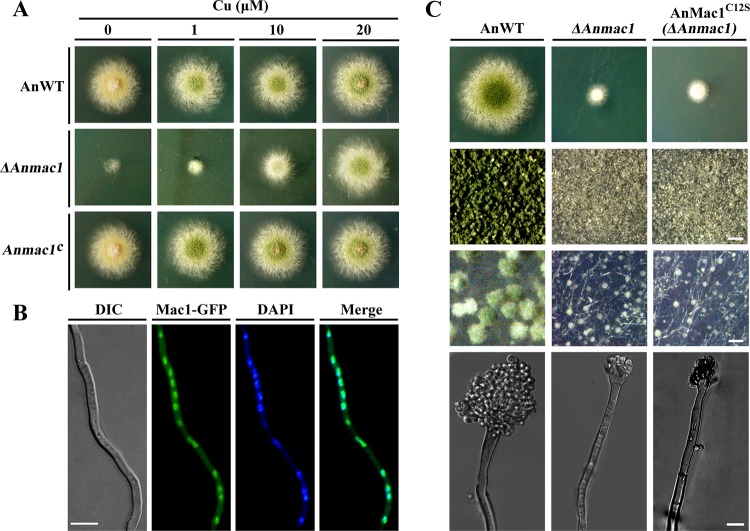
Phenotypic characterization of low-Cu-responsive transcription factor Mac1 in A. nidulans. (A) Equal numbers of conidia (2 × 10^4^) from the parental wild-type (WT) strain, *ΔAnmac1* mutant (*ΔAnmac1*) strain, and *Anmac1*-complemented (*Anmac1^c^*) strain were spot-inoculated onto MMPDR media supplemented with various concentrations of Cu, as indicated, and cultured at 37°C for 40 h for photography. (B) AnMac1 tagged with GFP (AnMac1-GFP) was ectopically expressed in the *ΔAnMac1* mutant. DIC microscopy and DAPI staining were used to visualize the hyphal morphologies and the nuclei, respectively. AnMac1-GFP localized to the nuclear region in A. nidulans. Scale bar = 10 μm. (C) Colony morphologies of the A. nidulans mutant strains (panel 1) grown on MMPDR media (1 μM Cu) at 37°C for 48 h. Closeup views of the center (panel 2) and edge (panel 3) of individual colonies were taken with a stereo microscope (bars represent 100 μm and 20 μm, respectively). Analyses of the conidiophore morphologies of the relevant mutant strains (panel 4) grown on MMPDR media (1 μM Cu) at 37°C for 40 h were performed. Scale bar = 10 μm.

As shown in Fig. 1, AnMac1 was able to functionally substitute AfMac1 for colony growth under conditions of Cu starvation. To further explore the function of Mac1 in Cu starvation in A. nidulans, we knocked out the ANIA_00658 gene in A. nidulans parental strain TN02A7 to generate a *ΔAnmac1* mutant. This was achieved by replacing the gene’s open reading frame (ORF) with the A. fumigatus *pyrG* marker, generating strain CZD05 (*ΔAnmac1*). Compared to the parental wild-type strain (A. nidulans WT [AnWT]), the *ΔAnmac1* mutant showed colonies with significantly reduced radial hyphal growth and fewer conidia under low-Cu conditions (Fig. 2A). This growth phenotype was rescued by the *Anmac1* gene, as demonstrated in *Anmac1*-complemented strain CZD06 (*Anmac1^c^*; Fig. 2A). As expected, exogenous Cu supplementation resulted in significant rescue of the defective phenotypes in the *ΔAnmac1* mutant in a dose-dependent manner, indicating that *Anmac1* regulates Cu uptake under low-Cu conditions. To further determine the cellular function of AnMac1, we generated a green fluorescent protein (GFP)-labeled AnMac1 strain (CZD07), expressing an AnMac1 C-terminal GFP-tagged fusion protein driven by an *AngpdA* constitutive promoter, in the *ΔAnmac1* mutant background. As expected, the AnMac1-GFP strain fully complemented the defects associated with loss of AnMac1 (Fig. S2), suggesting that the AnMac1-GFP fusion protein was functional. Importantly, cells expressing AnMac1-GFP showed strong nuclear localization signals, as demonstrated by DAPI (4′,6-diamidino-2-phenylindole)-stained nuclear distribution (Fig. 2B). The data suggest that AnMac1 is a nuclear localized transcription factor.

10.1128/mSphere.00670-18.2 FIG S2 Colony morphologies of Mac1-GFP strain grown on MMPDR media with 1 μM Cu at 37°C for 40 h. Download FIG S2, TIF file, 0.8 MB. Copyright © 2019 Cai et al. 2019 Cai et al. This content is distributed under the terms of the Creative Commons Attribution 4.0 International license.

Comparison of the colony morphologies of the WT and *ΔAnmac1* mutant strains revealed that the WT strain produced blue-green spores, unlike the *ΔAnmac1* mutant, which produced smaller, white conidia. Using a previously described sandwich coverslip protocol (31), we determined that vegetative mycelia of the WT strain developed into complete conidiophores with visible phialides connected by chains of numerous conidia, whereas the *ΔAnmac1* mutant produced severely abnormal structures of conidiophores with very few conidia. A phenotype similar to that of the *ΔAnmac1* mutant was observed in the AnMac1^C12S^ strain (Fig. 2C). These results imply that AnMac1-mediated Cu regulation is involved in the development of conidiophores and conidia.

### The Cu fist motif, but not the C-terminal Cys-rich motif, is required in Mac1 for its function in Cu starvation.

**FIG 3:**
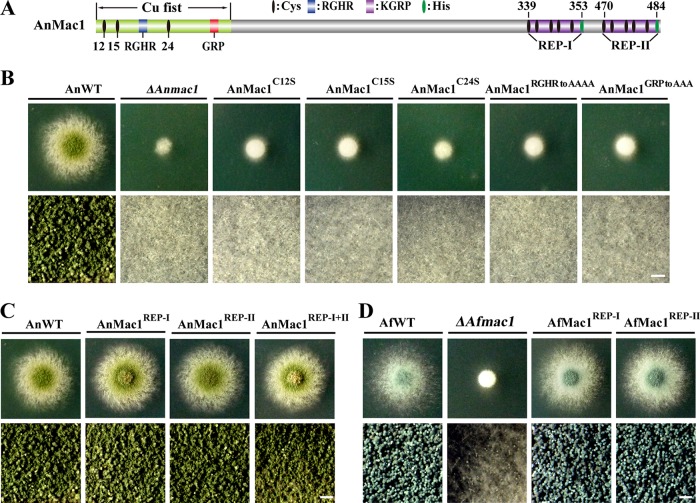
Molecular characterization of the key motifs of AnMac1. (A) Schematic view of the AnMac1 protein showing Cys, RGHR, and GRP residues in the N-terminal Cu fist structure as well as C-terminal Cys-rich motifs REP-I and REP-II. Black and green ovals indicate the positions of Cys and His residues, respectively. (B) Characterization of Cys, RGHR, and GRP mutants of A. nidulans grown on MMPDR media supplemented with 1 μM Cu at 37°C for 40 h. (C) Phenotypic characterization of the REP-I and REP-II A. nidulans mutant strains after growth on MMPDR media supplemented with 1 μM Cu at 37°C for 40 h. (D) A. fumigatus REP-I and REP-II mutant strains grown at 37°C for 40 h on 1 µM Cu-supplemented MM media. For panels B, C, and D, closeup views of the center of individual colonies were taken with a stereo microscope; the bars in those panels represent 100 μm.

To further assess the roles of the conserved Cys residue within the Cu fist domain, Cys residues at positions 12, 15, and 24 were individually mutated to Ser in the Cu fist domain of AnMac1 and were introduced into the *ΔAnmac1* background strain under the control of the *Anmac1* native promoter (Fig. 3A). The resulting AnMac1 mutant strains were referred to as CZD08 (AnMac1^C12S^), CZD09 (AnMac1^C15S^), and CZD10 (AnMac1^C24S^). In contrast to the WT strain, the three Cys mutants exhibited severe growth defects, with smaller colonies and fewer conidia, suggesting that these Cys residues are required for the normal function of AnMac1 under Cu starvation conditions (Fig. 3B). Similarly, by generating individual site-directed mutations, we found that conserved RGHR and GRP residues within the Cu fist domain are important for the AnMac1-mediated response to low-Cu conditions, as shown in strains CZD11 (AnMac1^RGHR to AAAA^) and CZD12 (AnMac1^GRP to AAA^) (Fig. 3B). These data suggest that the Cu fist motif harboring the conserved Cys, RGHR, and GRP residues is essential for Mac1 function in response to Cu starvation.

To ascertain the function of the conserved REP-I and REP-II motifs within the C terminus of AnMac1 (Fig. 1A and Fig. 3A), all of the Cys and His residues were mutated in each independent REP element. Unexpectedly, mutants AnMac1^REP-I^, AnMac1^REP-II^, and AnMac1^REP-I+II^ (CZD13, CZD14, and CZD15) all showed normal colony phenotypes comparable to those shown by the WT strain under Cu starvation conditions (Fig. 3C). This finding suggests that, under Cu starvation conditions, the C-terminal Cys-rich motifs are dispensable for AnMac1 function. Using the same strategy, we also demonstrated that the Cys-rich motifs are not required for AfMac1 function in Cu starvation in A. fumigatus, as shown by the AfMac1^REP-I^ and AfMac1^REP-II^ mutants (CZD16 and CZD17) (Fig. 3D). In summary, we conclude that N-terminal Cys residues play an important role in Mac1 function in the response of *Aspergillus* to low-Cu conditions, while the C-terminal Cys-rich motifs in AnMac1 are dispensable.

### Cu transporters CtrA2 and CtrC are both required for Mac1-mediated Cu uptake during Cu starvation in A. nidulans.

**FIG 4:**
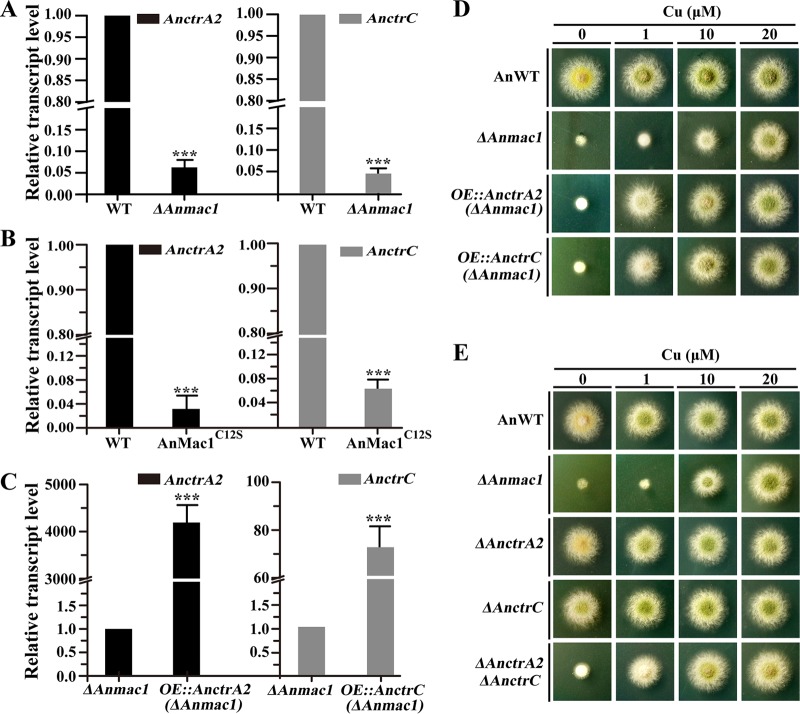
Expression and functional characterization of Cu transporters CtrA2 and CtrC in A. nidulans. (A) and (B) RT-qPCR analysis was performed after growth of cultures in liquid MMPDR media (1 μM Cu) for 18 h at 37°C, followed by a 1-h induction performed with 100 μM BCS. (C) RT-qPCR analysis was performed after growth of cultures in liquid MMPDR media (0 μM Cu) for 24 h at 37°C. For panels A, B, and C, the *tubA* gene was used as an internal control. ***, *P* < 0.001 (Student's *t* test); error bars represent standard deviations of results from three independent replicates. (D) Colony morphologies of *ΔAnmac1* mutant strains overexpressing *ctrA*2 and *ctrC* mutant strains grown on MMPDR media supplemented with various concentrations of Cu at 37°C for 40 h. (E) Effect of exogenous Cu supplementation on the colony morphologies of the *ΔAnctrA*2, *ΔAnctrC*, and *ΔAnctrA2 ΔAnctrC* mutants grown on MMPDR media at 37°C for 40 h.

Previously, we demonstrated that Mac1-mediated Cu uptake depends on transporters CtrA2 and CtrC in A. fumigatus (27). Using BLAST searches, we identified A. fumigatus *ctrA2* and *ctrC* homologs in A. nidulans as ANIA_03209 and ANIA_03813, here referred as *AnctrA2* and *AnctrC.* Furthermore, a predicted structure analysis showed that both AnCtrA2 and AnCtrC contain multiple transmembrane domains and Met-X_1–5_-Met motifs (Fig. S3A), which are typical signatures of the Cu transporters (32, 33). To elucidate the molecular mechanisms underlying the Mac1-mediated low-Cu response in A. nidulans, we examined the mRNA levels of *AnctrA2* and *AnctrC* in AnMac1 mutation strains. As shown in Fig. 4A and B, *AnctrA*2 and *AnctrC* were significantly downregulated in the *ΔAnmac1* and AnMac1^C12S^ mutants, indicating that *AnctrA2* and *AnctrC* were transcriptionally dependent on the presence of AnMac1. On the basis of real-time reverse transcription-quantitative PCR (RT-qPCR) data, we overexpressed *AnctrA*2 and *AnctrC* driven by the *AngpdA* constitutive promoter in the *ΔAnmac1* mutant. These strains were referred to as CZD18 and CZD19 (mutants *ΔAnmac1^OE^*^::^*^AnctrA^*^2^ and *ΔAnmac1^OE^*^::^*^AnctrC^*), respectively. Furthermore, we found that the expression levels of *AnctrA2* and *AnctrC* were significantly elevated in overexpression strains *ΔAnmac1^OE^*^::^*^AnctrA2^* and *ΔAnmac1^OE^*^::^*^AnctrC^*, respectively, compared to the level seen with the *ΔAnmac1* strain (Fig. 4C). These data suggest that *AnctrA2* and *AnctrC* were truly overexpressed but could not fully rescue defects induced by loss of *AnmacA*. As unpredicted, the strain overexpressing either *AnctrA*2 or *AnctrC* still displayed a defective colony phenotype upon Cu starvation (0 μM addition). Partial rescue of colony growth was achieved upon addition of Cu at a concentration of 1 or 10 μM (Fig. 4D). The data indicate that overexpression of AnCtrA2 or AnCtrC alone was unable to rescue the phenotype induced by the deletion of AnMac1 and that synergistic functioning of AnCtrA2 and AnCtrC seems to be required in the AnMac1-mediated Cu starvation response of the cell.

10.1128/mSphere.00670-18.3 FIG S3 Structural analysis of Ctr proteins. (A) A schematic representation of the predicted domains of CtrA2 and CtrC in A. nidulans and A. fumigatus. “M” represents methionine, and “X” represents any amino acid that is indicated with a number. The transmembrane domains (TM; indicated by a black-shaded box) were determined according to a TMHMM transmembrane analysis (http://www.cbs.dtu.dk/services/TMHMM/). (B) Alignment of AfCtrA2, AfCtrC, AnCtrA2, and AnCtrC using the AlignX module of Vector NTI Advance software. Download FIG S3, TIF file, 0.6 MB. Copyright © 2019 Cai et al. 2019 Cai et al. This content is distributed under the terms of the Creative Commons Attribution 4.0 International license.

To further illuminate the function of *AnctrA*2 and *AnctrC* in response to low-Cu environments, we constructed single and double deletions of *AnctrA2* and *AnctrC*. As seen in Fig. 4E, single deletion mutants *ΔAnctrA2* and *ΔAnctrC* (CZD20 and CZD21) exhibited WT-like colony morphologies, whereas the double deletion mutant, *ΔAnctrA2 ΔAnctrC* (CZD22), showed severe defects in low-Cu environments, especially under Cu starvation conditions. Notably, addition of exogenous Cu to the media (at 1 and 10 μM Cu) rescued the defective phenotypes of the *ΔAnctrA2 ΔAnctrC* double deletion strain. In comparison, 1 μM Cu was unable to rescue the colony defects in a *ΔAnmac1* mutant, whereas phenotype rescue was seen at 10 µM and 20 µM. Thus, the data suggest that higher concentrations of Cu addition can bypass the requirement of the AnMac1-Ctr pathway and that Mac1 plays a more important role in Cu uptake than regulatory Cu transporters CtrA2 and CtrC in A. nidulans.

## DISCUSSION

Copper acquisition is important for cell survival and proliferation, especially during periods of Cu starvation. The low-Cu-responsive transcription factor Mac1 and its regulated Cu transporters have been reported in A. fumigatus. However, the key motif(s) by which Mac1 senses Cu has not been defined and is important for understanding how Cu uptake is controlled in Aspergillus. In this study, site-directed-mutagenesis experiments, combined with homolog complementation assays, demonstrated that the Cu fist motif is essential, but not sufficient, for AnMac1-mediated Cu uptake during Cu starvation. Our findings may have broad implications for the structurally conserved Mac1 homologs in *Aspergillus*. In addition, unlike their counterparts in A. fumigatus (27), overexpression of the transporters AnCtrA2 and AnCtrC cannot functionally compensate for the loss of AnMac1 under the low-Cu culture conditions. Further, our data suggest that AnCtrC is responsible for low-Cu response only in the absence of AnCtrA2, indicating that the conserved Mac1-mediated Cu uptake in A. fumigatus and A. nidulans also possesses species-specific machinery.

Our data further demonstrated that A. nidulans Mac1 could functionally cross-complement A. fumigatus Mac1 deletion in response to low-Cu conditions, indicating that the Cu uptake mechanism may be conserved in Aspergillus. However, there was a significant difference in the threshold value of Cu required for the phenotype rescue. Addition of 20 μM Cu sulfate in minimal media resulted in an almost complete restoration of the WT phenotype in the *ΔAnmac1* mutant, whereas the *ΔAfmac1* mutant required more than 100 μM Cu to be rescued. This finding suggests that, in addition to Mac1, A. nidulans may have other high-affinity Cu-uptake systems to bypass the requirement of AnMac1, while A. fumigatus may not (27). Importantly, overexpression of either Cu transporter AnCtrA2 or Cu transporter AnCtrC in A. nidulans failed to completely restore the Δ*Anmac1* mutant to the WT phenotype during Cu starvation. This implies that, in contrast to A. fumigatus (27), in the absence of AnMac1, Cu transporters AnCtrA2 and AnCtrC may need to function together. Another possibility is that there might be some unknown AnMac1-mediated targets that also play a role in the Cu uptake process in A. nidulans. In addition, we demonstrated that the CtrC Cu transporter in A. fumigatus, but not that in A. nidulans, is responsible for the Cu starvation response. Furthermore, the alignment analysis showed that the level of amino acid sequence identity of AnCtrC and AfCtrC was 45.3%, while AnCtrA2 showed only 11% sequence identity with AfCtrA2 but 34.5% sequence identity with AnCtrC (see Fig. S3B in the supplemental material). The result suggests that the CtrC Cu transporter but not CtrA2 possess high conservation between A. nidulans and A. fumigatus, implying that CtrA2 may have different functions for Cu uptake in A. nidulans and A. fumigatus. Collectively, these results suggest that the conserved Mac1-mediated Cu uptake in A. fumigatus and that in A. nidulans also have their own unique styles.

In S. cerevisiae, both Cu fist structures and Cys-rich motifs are involved in Cu-dependent DNA binding, indicating that they are potentially important for Mac1 function in *Aspergillus*. Site-directed mutagenesis demonstrated that the conserved Cu fist motif is responsible for the AnMac1-mediated Cu starvation response. Furthermore, data from site-directed mutagenesis and homolog replacement experiments led us to conclude that the conserved Cu fist domain is essential, but not sufficient, for the AfMac1-mediated functional response to low-Cu conditions. This indicated that the conserved Cu fist motif requires cooperation with nonconserved regions to accomplish Mac1-mediated low-Cu response in Aspergillus. Interestingly, the Cu fist domain is conserved among the members of the family of Cu-responsive transcription factors (27, 34). We have previously demonstrated that the Cu fist motif is required for the function of the high-Cu-sensing transcription factor AceA in Cu detoxification in A. fumigatus (34). Therefore, both high-Cu-sensing and low-Cu-sensing transcription factors may utilize a common mechanism to bind DNA in A. nidulans *and* A. fumigatus. However, in S. cerevisiae, the Cu detoxification transcription factor Ace1 utilizes N-terminal Cys-X_1-2_-Cys motifs to bind Cu^+^ ions in a polycopper cluster, in contrast to the C-terminal Cys-rich motifs in REP-I and REP-II in Mac1 (13, 16, 20, 21). This difference implies that high-Cu-sensing and low-Cu-sensing transcription factors may have distinct Cu-binding domains in yeasts. Notably, we found that the REP-I and REP-II motifs are dispensable for Mac1 function in Cu starvation in both A. nidulans and A. fumigatus, suggesting that they possess Cu-ion binding characteristics that are distinct from those possessed by yeasts. However, we still cannot exclude the possibility that REP-I and REP-II motifs may play a role in Mac1 function in high-Cu-induced degradation of Cu transporters analogous to the role seen in S. cerevisiae (21).

## MATERIALS AND METHODS

### Strains, oligonucleotides, media, and transformation.

Lists of all the *Aspergillus* strains and oligonucleotides used in this study are provided in Table S1 and Table S2, respectively. The TN02A7 deletion strain of a gene required for nonhomologous end joining in double-strand break repair (35, 36) was used to generate the *ΔAnmac1*, *ΔAnctrA2*, and *ΔAnctrC* mutant strains. All A. fumigatus strains were grown on minimal MM solid medium (1% glucose, 1 ml liter^−1^ trace elements, 50 ml liter^−1^ 20× salt [pH 6.5], and 2% agar), while all of the A. nidulans strains were grown on minimal MMPDR solid medium (2% glucose, 1 ml liter^−1^ trace elements, 50 ml liter^−1^ 20× salt, 0.5 mg liter^−1^ pyridoxine, 2.5 mg liter^−1^ riboflavin, 2% agar, pH 6.5) (27, 37, 38). The agar was omitted for liquid medium. Notably, the content of CuSO_4_ was removed from the trace elements as previously described (39), with a few modifications, such that Cu was absent in both the MM and the MMPDR media used in this study. Transformation was performed following published protocols (40, 41).

10.1128/mSphere.00670-18.4 TABLE S1 *Aspergillus* strains used in this study. Download Table S1, DOCX file, 0.02 MB. Copyright © 2019 Cai et al. 2019 Cai et al. This content is distributed under the terms of the Creative Commons Attribution 4.0 International license.

10.1128/mSphere.00670-18.5 TABLE S2 Primers used in this study. Download Table S2, DOCX file, 0.02 MB. Copyright © 2019 Cai et al. 2019 Cai et al. This content is distributed under the terms of the Creative Commons Attribution 4.0 International license.

### Construction of strains for AnMac1 homolog replacement experiments.

To construct an *Anmac1* open reading frame (ORF) driven by the *Afmac1* native promoter, fragments containing regions flanking the 5′ and 3′ *Afmac1* ORF were amplified from A. fumigatus A1160 genomic DNA with primer pairs Afmac1(p)-F/Afmac1(p)-An-R and Afmac1(t)-F/Afmac1(t)-R. The coding sequence of AnMac1 ORF was amplified with primer pair Anmac1(ORF)-S/Anmac1(ORF)-A. The resulting three PCR products were cloned into plasmid pAN7-1 to generate plasmid pCD01 (*ΔAfmac1*^AnMac1^). A similar strategy was used to construct plasmids pCD02 (*ΔAfmac1*^ScMac1^) and pCD03 (*ΔAfmac1*^SpCuf1^). The *Afmac1* homolog complemented strains (CZD01 to CZD03) were generated by individually transforming plasmids pCD01 to pCD03 into the *ΔAfmac1* mutant. Using plasmid pCD01 as a template, the two DNA fragments amplified with primer pairs Afmac1(p)-F/AnMac1^C12S^-R and AnMac1^C12S^-F/Afmac1(t)-R, respectively, were cloned into plasmid pAN7-1 to generate plasmid pCD04. Strain CZD04 [*ΔAfmac1*^AnMac1(C12S)^] was generated by transforming plasmid pCD04 into the *ΔAfmac1* mutant.

### Construction of gene deletion and complemented strains.

The *Anmac1* gene was deleted in the parental A. nidulans TN02A7 strain. The *pyrG* selectable marker from A. fumigatus was amplified from plasmid pFNO3 with primer pair AfpyrG-F/AfpyrG-R. The 5′ and 3′ flanking regions of the *Anmac1* gene were amplified from A. nidulans TN02A7 genomic DNA with primer pairs Anmac1-P2/Anmac1-P3 and Anmac1-P4/Anmac1-P5, respectively. The resulting DNA fragments were cloned into plasmid pUC19 using a ClonExpress MultiS One Step cloning kit (Vazyme; C113-02), and the product was then transformed into the TN02A7 strain to generate the CZD05 strain. The *ΔAnmac1* transformants were confirmed by diagnostic PCR using *Anmac1* ORF primer pair Anmac1-S/Anmac1-A and primer pairs Anmac1-P1/R-pyrG and Anmac1-P6/F-pyrG. A similar strategy was used to construct mutant strains *ΔAnctrA2* (CZD20) and *ΔAnctrC* (CZD21). In addition, double mutant *ΔAnctrA2 ΔAnctrC* (CZD22) was generated by cotransforming both *AnctrA2* and *AnctrC* deletion constructs into the TN02A7 strain. The *Anmac1*-complemented fragments and a selectable nutritional marker, *pyroA*, amplified with primer pairs Anmac1-P7/Anmac1-P8 and pyroA-F/pyroA-pUC19-R, respectively, were cloned into plasmid pUC19. The *Anmac1*-complemented strain (CZD06) was generated by transforming complementation plasmid pCD05 into the *ΔAnmac1* mutant background.

### Construction of GFP-labeled strain AnMac1-GFP.

We generated strain CZD07 (AnMac1-GFP), which expresses AnMac1 transcriptionally fused with a GFP tag at its C terminus under the control of the *AngpdA* promoter. To achieve this, we ﬁrst ampliﬁed AnMac1 (without the termination codon) and GFP using primer pairs AnMac1-GFP-S/AnMac1-GFP-A and gfp-F/gfp-R, respectively. The two fragments were then cloned into reconstituted plasmid vector pBARGPE1, which contains the *AngpdA* promoter, the *trpC* terminator, and the *pyroA* selectable nutritional marker. After transformation into the *ΔAnmac1* mutant was performed, we generated the GFP-labeled CZD07 strain (AnMac1-GFP).

### Construction of strains overexpressing *AnctrA2* and *AnctrC*.

Primer pair OE::AnctrA2-F/OE::AnctrA2-R was used to amplify the *AnctrA2* ORF, which was then cloned into reconstituted plasmid vector pBARGPE1, which contains the *AngpdA* promoter, the *trpC* terminator, and the *pyroA* selectable nutritional marker. After transformation into the *ΔAnmac1* mutant was performed, we generated *AnctrA2* overexpression strain CZD18 (*ΔAnmac1^OE^*^::^*^AnctrA2^*). The same strategy was used to generate *AnctrC* overexpression strain CZD19 (*ΔAnmac1^OE^*^::^*^AnctrC^*).

### Construction of Mac1-related point mutation strains.

AnMac1 reconstruction plasmid pCD06, carrying a mutation of Cys at position 12, was constructed as follows: using plasmid pCD05 as a template, two DNA fragments amplified with primer pairs AnMac1-P7/AnMac1^C12S^-R and AnMac1^C12S^-F/pyroA-pUC19-R, respectively, were cloned into plasmid pAN7-1 to generate plasmid pCD06 (AnMac1^C12S^). Similar strategies were used to construct plasmids carrying additional AnMac1 mutations, such as pCD07 (AnMac1^C15S^), pCD08 (AnMac1^C24S^), pCD09 (AnMac1^RGHR to AAAA^), pCD10 (AnMac1^GRP to AAA^), pCD11 (REP-I, AnMac1^C339S, C341S, C345S, C347S, C350S, and H353G^), and pCD12 (REP-II, AnMac1^C470S, C472S, C476S, C478S C481S, and H484G^). Using plasmid pCD11 as a template, two DNA fragments amplified with primer pairs AnMac1-P7/AnREP-II-R and AnREP-II-F/pyroA-pUC19-R, respectively, were cloned into plasmid pAN7-1 to generate plasmid pCD13 (AnMac1^REP-I+REP-II^). Mutant strains CZD08 to CZD15 were generated by transforming plasmids pCD06 to pCD13 into the *ΔAnmac1* mutant. Similarly, we generated the two REP-I and REP-II mutants (CZD16 and CZD17) in the A. fumigatus *ΔAfmac1* mutant.

### Microscopic observation and image processing.

For hyphal microscopy, conidia were inoculated onto precleaned glass coverslips overlaid with MMPDR liquid media. The strains were grown on coverslips at 37°C for various durations prior to observation under a microscope. The DNA was stained using 4,6-diamidino-2-phenylindole (DAPI) after fixing the cells with 4% paraformaldehyde (Polyscience, Warrington, PA) (42, 43). Differential interference contrast (DIC) images and fluorescent images of the cells were collected using a Zeiss Axio imager A1 microscope (Zeiss, Jena, Germany).

For observation of conidiophore structure, the slide culture method for microscopic observation was performed as previously described (31, 44), with a few modifications. Conidia were inoculated on the edge of a small square of agar medium placed on top of a coverslip, which was placed in a petri dish containing solidiﬁed agar to keep it moist. Another coverslip was placed on top of the agar square after inoculation. The coverslips with aerial hyphae and attached conidiophores were imaged using a SensiCam QE cooled digital camera system (Cooke Corporation, Germany) and analyzed with the MetaMorph/MetaFluor combination software package (Universal Imaging, West Chester, PA).

### RNA extraction and semiquantitative RT-PCR or RT-qPCR analysis.

A.nidulans conidia were inoculated into liquid MMPDR media with 1 μM Cu and shaken on a rotary shaker at 220 rpm and 37°C for 18 h, followed by a subsequent 1-h induction with 100 μM Cu chelator bathocuproine disulfonic acid (BCS). The mycelia were ground in liquid nitrogen. Total RNA was isolated using TRIzol (Invitrogen; 15596-025) following the instructions of the manufacturer. One hundred milligrams of mycelia per sample was used as the starting material for the determination of total RNA. RT-qPCR experiments were carried out using HiScript Q RT SuperMix for qPCR (Vazyme; R123-01) (with a genomic DNA [gDNA] wiper), and then cDNA was used as the template for testing the transcripts of the relevant genes. For RT-qPCR analysis, the threshold cycle (2^−Δ^*^CT^*) method was used to determine the expression level. For semiquantitative RT-PCR analysis, A. fumigatus conidia were inoculated into liquid MM media with 0 μM Cu and shaken on a rotary shaker at 220 rpm and 37°C for 24 h. All data were obtained on the basis of results from three independent experiments.
