# Supplementary figures and images for: Molecular Characteristics of the Conserved Aspergillus nidulans Transcription Factor Mac1 and Its Functions in Response to Copper Starvation
Source: mSphere. 2019 Jan 30;4(1):e00670-18. doi: 10.1128/mSphere.00670-18 (PMC6354809; doi:10.1128/mSphere.00670-18)

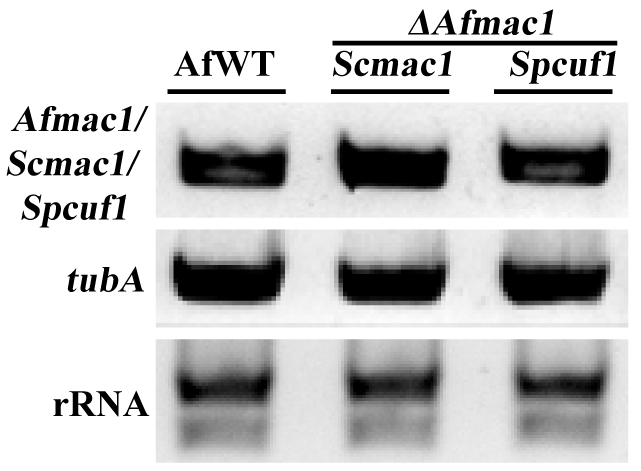

Supplement: FIG S1 [file mSphere.00670-18-sf001.tif]

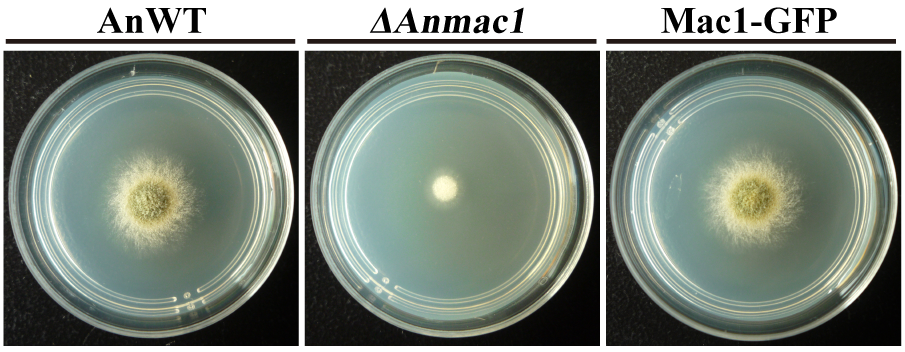

Supplement: FIG S2 [file mSphere.00670-18-sf002.tif]

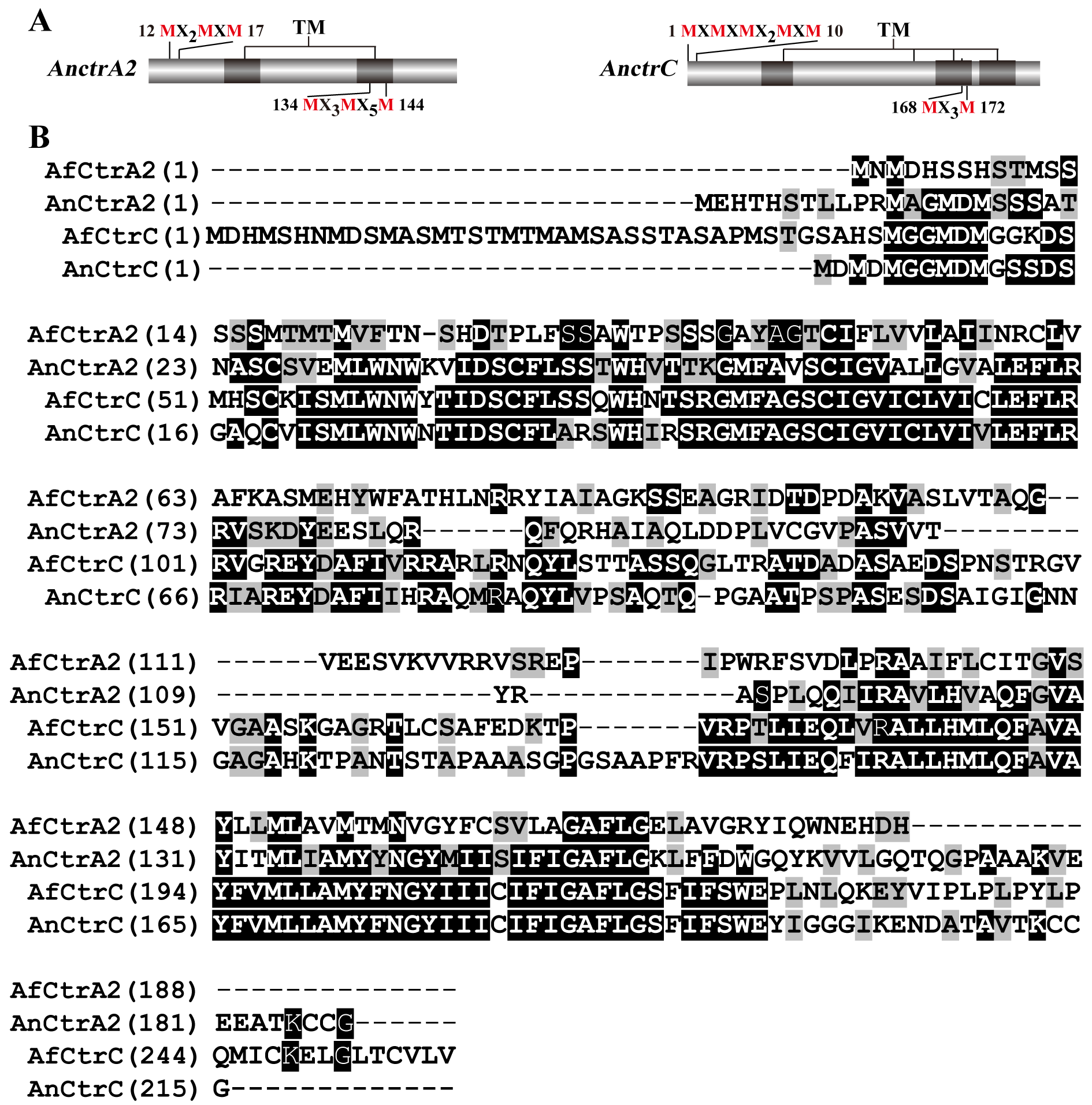

Supplement: FIG S3 [file mSphere.00670-18-sf003.tif]
