# Supplementary material for: Molecular Characteristics of the Conserved Aspergillus nidulans Transcription Factor Mac1 and Its Functions in Response to Copper Starvation
Source: mSphere. 2019 Jan 30;4(1):e00670-18. doi: 10.1128/mSphere.00670-18 (PMC6354809; doi:10.1128/mSphere.00670-18)
Supplement: TABLE S1 [file mSphere.00670-18-st001.docx]

| **Table S1** *Aspergillus* strains used in this study. | | |
| --- | --- | --- |
| Strain | Genotype | Reference or source |
| AfWT | *Δku80*; A1160::*pyrG1* | (1) |
| *ΔAfmac1* | *Δku80; pyrG1; ΔAfmac1::pyr4; veA1* | (1) |
| TN02A7 | *pyrG89; riboB2; nkuA::argB2; pyroA4* | (2) |
| CZD01 | *Δku80; pyrG1; ΔAfmac1::pyr4; Afmac1 (p)::Anmac1::hph; veA1* | This study |
| CZD02 | *Δku80; pyrG1; ΔAfmac1::pyr4; Afmac1 (p)::Scmac1::hph; veA1* | This study |
| CZD03 | *Δku80; pyrG1; ΔAfmac1::pyr4; Afmac1 (p)::Afmac1^(Spcuf1)^::hph; veA1* | This study |
| CZD04 | *Δku80; pyrG1; ΔAfmac1::pyr4; Afmac1 (p)::Anmac1^(C12S)^::hph; veA1* | This study |
| CZD05 | *ΔAnmac1*::*pyrG; pyrG89; riboB2; nkuA::argB2; pyroA4* | This study |
| CZD06 | *ΔAnmac1*::*pyrG; Anmac1(p)::Anmac1::pyroA; pyrG89; riboB2; nkuA::argB2; pyroA4* | This study |
| CZD07 | *ΔAnmac1::pyrG; AngpdA(p)::Anmac1*-GFP*::pyroA; pyrG89; riboB2; nkuA::argB2; pyroA4* | This study |
| CZD08 | *ΔAnmac1*::*pyrG; Anmac1(p)::Anmac1^(C12S)^::pyroA; pyrG89; riboB2; nkuA::argB2; pyroA4* | This study |
| CZD09 | *ΔAnmac1*::*pyrG; Anmac1(p)::Anmac1^(C15S)^::pyroA; pyrG89; riboB2; nkuA::argB2; pyroA4* | This study |
| CZD10 | *ΔAnmac1*::*pyrG; Anmac1(p)::Anmac1^(C24S)^::pyroA; pyrG89; riboB2; nkuA::argB2; pyroA4* | This study |
| CZD11 | *ΔAnmac1*::*pyrG; Anmac1(p)::Anmac1^(RGHR to AAAA)^::pyroA; pyrG89; riboB2; nkuA::argB2; pyroA4* | This study |
| CZD12 | *ΔAnmac1*::*pyrG; Anmac1(p)::Anmac1^(GRP to AAA)^::pyroA; pyrG89; riboB2; nkuA::argB2; pyroA4* | This study |
| CZD13 | *ΔAnmac1*::*pyrG; Anmac1(p)::Anmac1^(REP-Ⅰ)^::pyroA; pyrG89; riboB2; nkuA::argB2; pyroA4* | This study |
| CZD14 | *ΔAnmac1*::*pyrG; Anmac1(p)::Anmac1^(REP-Ⅱ)^::pyroA;pyrG89; riboB2; nkuA::argB2; pyroA4* | This study |
| CZD15 | *ΔAnmac1*::*pyrG; Anmac1(p)::Anmac1^(REP-Ⅰ^*^+^*^Ⅱ)^::pyroA; pyrG89; riboB2; nkuA::argB2; pyroA4* | This study |
| CZD16 | *Δku80; pyrG1; ΔAfmac1::pyr4;* *Afmac1 (p)::Afmac1^(REP-Ⅰ)^::hph; veA1* | This study |
| CZD17 | *Δku80; pyrG1; ΔAfmac1::pyr4; Afmac1 (p)::Afmac1^(REP-Ⅱ)^::hph; veA1* | This study |
| CZD18 | *ΔAnmac1::pyrG; AngpdA(p)::AnctrA2::pyroA; pyrG89; riboB2; nkuA::argB2; pyroA4* | This study |
| CZD19 | *ΔAnmac1::pyrG; AngpdA(p)::AnctrC::pyroA; pyrG89; riboB2; nkuA::argB2; pyroA4* | This study |
| CZD20 | *ΔAnctrA2*::*pyrG; pyrG89; riboB2; nkuA::argB2; pyroA4* | This study |
| CZD21 | *ΔAnctrC::pyroA; pyrG89; riboB2; nkuA::argB2; pyroA4* | This study |
| CZD22 | *ΔAnctrA2*::*pyrG; ΔAnctrC::pyroA; pyrG89; riboB2; nkuA::argB2; pyroA4* | This study |

1. Cai Z, Du W, Zeng Q, Long N, Dai C, Lu L. 2017. Cu-sensing transcription factor Mac1 coordinates with the Ctr transporter family to regulate Cu acquisition and virulence in *Aspergillus fumigatus*. Fungal Genet Biol 107:31-43.

2. Zhong GW, Wei WF, Guan Q, Ma ZF, Wei H, Xu XS, Zhang SZ, Lu L. 2012. Phosphoribosyl pyrophosphate synthetase, as a suppressor of the sepH mutation in *Aspergillus nidulans*, is required for the proper timing of septation. Mol Microbiol 86:894-907.
