# Supplementary material for: Molecular Characteristics of the Conserved Aspergillus nidulans Transcription Factor Mac1 and Its Functions in Response to Copper Starvation
Source: mSphere. 2019 Jan 30;4(1):e00670-18. doi: 10.1128/mSphere.00670-18 (PMC6354809; doi:10.1128/mSphere.00670-18)
Supplement: TABLE S2 [file mSphere.00670-18-st002.docx]

| **Table S2** Primers used in this study | |
| --- | --- |
| Primer/Purpose | Sequence (5’ - 3’ direction) |
| ***Anmac1* deletion and complementation** | |
| Anmac1-P1 | TTCTATTCATACCTGCCACTT |
| Anmac1-P2 | AAACGACGGCCAGTGAATTCGGTATGTGAGCAAGGGAAAG |
| Anmac1-P3 | GAAGAGCATTGTTTGAGGCGGGCGAGTATACAGATTGGCG |
| Anmac1-P4 | ATTTTATCCATTTGAGTGG |
| Anmac1-P5 | GACCATGATTACGCCAAGCTTCTTGGTCCATTCGCTGTT |
| Anmac1-P6 | GGACCCGAAGCTCGAAAA |
| Anmac1-P7 | AAACGACGG CCAGTGAATTCGGTATGTGAGCAAGGGAAAG |
| Anmac1-P8 | CTATTATCTGACTTACCCGCCATAATGTAGCCTCTTTCC |
| Anmac1-S | ATGCCTTTCGACGAAAACGG |
| Anmac1-A | GGTTGGATTTATGGGTGA |
| F-pyrG | TTCGTTATGGGTTTCGTGTC |
| R-pyrG | CAGGCAGAACTGTAGAAG |
| AfpyrG-F | CGCCTCAAACAATGCTCTTC |
| Anmac1-AfpyrG-R | CCACTCAAATGGATAAAATCTGTCTGAGAGGAGGCACTGATG |
| pyroA-F | GCGGGTAAGTCAGATAATAG |
| pyroA-pUC19-R | GACCATGATTACGCCAAGCTTCTGACTTGACGCTTTCTCTTGG |
| **AnctrA2 deletion** | |
| AnctrA2-P1 | AGCCGAGCCAGCGGTGTAAG |
| AnctrA2-P2 | AAACGACGGCCAGTGAATTCCCGACAAAAGTGAAGACCGT |
| AnctrA2-P3 | GAAGAGCATTGTTTGAGGCGCTTGTCGAATGTCTGGATAC |
| AnctrA2-P4 | GTGGCTAGCTTTGCTGTAT |
| AnctrA2-P5 | GACCATGATTACGCCAAGCTTCTGAGTCCCTCGGGTATTGC |
| AnctrA2-P6 | CGGGCACAAGAATGCTGACT |
| AnctrA2-S | ATGGAGCACACACACTCAAC |
| AnctrA2-A | AACACTTGGTCGCTTCCTCC |
| F-pyrG | TTCGTTATGGGTTTCGTGTC |
| R-pyrG | CAGGCAGAACTGTAGAAG |
| AfpyrG-F | CGCCTCAAACAATGCTCTTC |
| AnctrA2-AfpyrG-R | ATACAGCAAAGCTAGCCACCTGTCTGAGAGGAGGCACTGATG |
| **AnctrC deletion** | |
| AnctrC-P1 | CGATGAACGACACCCTGGAC |
| AnctrC-P2 | AAACGACGGCCAGTGAATTCTAGGCGTGGATGGGTGTAAG |
| AnctrC-P3 | CTATTATCTGACTTACCCGCCTTGTTAATGTCGTTTGTTGC |
| AnctrC-P4 | GGTTGATGTTTCACATACGC |
| AnctrC-P5 | GACCATGATTACGCCAAGCTTTGTTCTGAAGGCTAACTATG |
| AnctrC-P6 | ACCTTCCACCGTATTATCCC |
| AnctrC-S | ATGGACATGGACATGGGAGG |
| AnctrC-A | GCAGCATTTCGTAACAGCCG |
| pyroA-F | GCGGGTAAGTCAGATAATAG |
| AnctrC-pyroA-R | GCGTATGTGAAACATCAACCCTGACTTGACGCTTTCTCTTGG |
| F-PyroA | AGGCTGGCATCAGACATTC |
| R-PyroA | AACATCCATAATAACACCGC |
| **AnMac1 mutation** | |
| AnMac1^C12S^-R | CTTCAAGTTGTTACACCGCGTAATGCTTACGAAGACC |
| AnMac1^C12S^-F | GCGGTGTAACAACTTGAAG |
| AnMac1^C15S^-R | GTGACCGCGAATGGATGGCTCACTGGATTGGAGTCC |
| AnMac1^C15S^-F | CCATCCATTCGCGGTCAC |
| AnMac1^C24S^-R | CATAATGGGCGGATTTGGACGAACGGTGACCGCGAATG |
| AnMac1^C24S^-F | GTCCAAATCCGCCCATTATG |
| AnMac1^RGHR to AAAA^-R | GCGCATTTGGACGAAGCGGCAGCGGCAATGCATGGCTCAC |
| AnMac1^RGHR to AAAA^-F | GCCGCTTCGTCCAAATGCGC |
| AnMac1^AGRP to AAA^-R | GACACTTAGACAGCGCGGCCGCGGCTTTTCCGACGCTCACC |
| AnMac1^AGRP to AAA^-F | GCGGCCGCGCTGTCTAAGTGTC |
| AnREP-I-R | CGGCTCGTCCGGGCTAGCCAAGCTACTGCTTTGAGGGCCACTACTGCTGCTATGG |
| AnREP-I-F | AGTAGCTTGGCTAGCCCGGACGAGCCG |
| AnREP-II-R | GCCCTCTGTTAAACTGCCAAGGCTGCTACTGCTGGGACCACTTTGGCTATTACCG |
| AnREP-II-F | AGCAGCCTTGGCAGTTTAACAGAGGGC |
| AfREP-I-R | GCTTCCTAGACTCTGGCTACTGTCCCCACTGTTGCTGTCATGC |
| AfREP-I-F | CAGTAGCCAGAGTCTAGGAAGC |
| AfREP-II-R | GACTGCCAACGCTGGCACTATCGTTGCCGCTGAGGCTACTGC |
| AfREP-II-F | GATAGTGCCAGCGTTGGCAGTC |
| **AnMac1 homolog replacement** | |
| Afmac1(p)-F | ACCTGCAGGCATGCAAGCTTGATGACGGAGGATGGGAGAG |
| Afmac1(p)-An-R | CCGTTTTCGTCGAAAGGCATGCTGGCAGGCACCTGATATAC |
| Anmac1(ORF)-S | ATGCCTTTCGACGAAAACGG |
| Anmac1(ORF)-A | CACCATCAATCGCTAAATCTCTAAGTAAACCAGCAACCAG |
| Afmac1(t)-F | AGATTTAGCGATTGATGGTG |
| Afmac1(t)-R | CGACGGCCAGTGCCAAGCTTCATGTACGATACCTGCATAGG |
| Afmac1(p)-Sc-R | GTTCCCATTAAATATTATCATGCTGGCAGGCACCTGATATAC |
| Scmac1(ORF)-S | ATGATAATATTTAATGGGAAC |
| Scmac1(ORF)-A | CACCATCAATCGCTAAATCTTTATGAAGTGGTGGCATCGC |
| Afmac1(p)-Sp-R | CGTTATTGATTACAACCATGCTGGCAGGCACCTGATATAC |
| Spcuf1(ORF)-S | ATGGTTGTAATCAATAACG |
| Spcuf1(ORF)-A | CACCATCAATCGCTAAATCTTCATGTTGAACCTGTTGTAA |
| **Over-expression** | |
| OE::AnctrA2-F | GGCTGCAGGAATTCGATATCATGGAGCACACACACTCAAC |
| OE::AnctrA2-R | GTATCGATAAGCTTGATATCCTAGCCACAACACTTGGTCG |
| OE::AnctrB-F | GGCTGCAGGAATTCGATATCATGGACATGTACCATATGGG |
| OE::AnctrB-R | GTATCGATAAGCTTGATATCTCAATGACAAGCGACCGAC |
| **AnMac1-GFP** | |
| AnMac1-GFP-S | GGCTGCAGGAATTCGATATCATGCCTTTCGACGAAAACGG |
| AnMac1-GFP-A | CCAGCGCCTGCACCAGCTCCCAGATTGCCGTGTGTTAAAC |
| gfp-F | GGAGCTGGTGCAGGCGCTGG |
| gfp-R | GTATCGATAAGCTTGATATCTTTGTATAGTTCATCCATGCC |
| **RT-PCR** | |
| Afmac1-F | GAGTATCCCTCAGAGCAAC |
| Afmac1-R | GCATTGGGTTTGGTGATTGA |
| Scmac1-F | CATAGAAGCGAAGAGGAAC |
| Scmac1-R | AATATTTTGACGGCACCA |
| Spcuf1-F | GCTATGCTGGCAGATGGA |
| Spcuf1-R | TTCGTGCTTGAAAGTATGATGG |
| AnctrA2-F | GCGTTCTTGGGCAAACTCTT |
| AnctrA2-R | CACTTGGTCGCTTCCTCCTC |
| AnctrC-F | AGATGCGTGCTCAATACCTT |
| AnctrC-R | GGTGTTGGCAGGAGTCTTAT |
